# 3-Phenyl-*N*,*N*,*N*′,*N*′-tetra­methyl-1-ethyne-1-carboximidamidium bromide

**DOI:** 10.1107/S1600536812021873

**Published:** 2012-05-19

**Authors:** Ioannis Tiritiris, Willi Kantlehner

**Affiliations:** a Institut für Organische Chemie, Universität Stuttgart, Pfaffenwaldring 55, 70569 Stuttgart, Germany; b Fakultät Chemie/Organische Chemie, Hochschule Aalen, Beethovenstrasse 1, D-73430 Aalen, Germany

## Abstract

The reaction of 3,3,3-tris­(dimethyl­amino)-1-phenyl­prop-1-yne with bromine in pentane yields the title compound, C_13_H_17_N_2_^+^·Br^−^. The acetyl­enic bond distance [1.197 (2) Å] is consistent with a C C triple bond. The amidinium C=N bonds [1.325 (2) and 1.330 (2) Å] have double-bond character and the positive charge is delocalized between the two dimethyl­amino groups.

## Related literature

For the synthesis of alkynyl orthoamides and acetyl­enic amidinium salts, see: Weingärtner *et al.* (2011 ▶). For the synthesis of vinyl­ogous guanidinium iodides and bromides, see: Kantlehner *et al.* (2012 ▶). For the crystal structure of *N*,*N*,*N*′,*N*′,*N*′′,*N*′′,*N*′′′,*N*′′′-octa­methyl-(but-2-yne)-bis­(amidinium)-bis­(tetra­fluoridoborate), see: Drandarov *et al.* (2012 ▶).
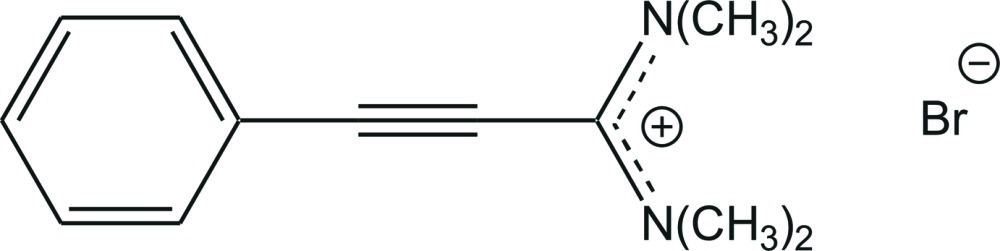


## Experimental

### 

#### Crystal data

C_13_H_17_N_2_^+^·Br^−^
*M* *_r_* = 281.19
Monoclinic, 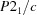

*a* = 13.1009 (8) Å
*b* = 10.6538 (6) Å
*c* = 9.6611 (6) Å
β = 100.276 (3)°
*V* = 1326.81 (14) Å^3^
*Z* = 4
Mo *K*α radiation
μ = 3.08 mm^−1^
*T* = 100 K
0.28 × 0.20 × 0.15 mm

#### Data collection

Bruker Kappa APEXII DUO diffractometer
Absorption correction: multi-scan (Blessing, 1995) ▶ *T*_min_ = 0.483, *T*_max_ = 0.630
27687 measured reflections
4075 independent reflections
3466 reflections with *I* > 2σ(*I*)
*R*_int_ = 0.032

#### Refinement

*R*[*F* ^2^ > 2σ(*F* ^2^)] = 0.021
*wR*(*F* ^2^) = 0.052
*S* = 1.07
4075 reflections
149 parameters
H-atom parameters constrained
Δρ_max_ = 0.38 e Å^−3^
Δρ_min_ = −0.43 e Å^−3^

### 

Data collection: *APEX2* (Bruker, 2008 ▶); cell refinement: *SAINT* (Bruker, 2008 ▶); data reduction: *SAINT*; program(s) used to solve structure: *SHELXS97* (Sheldrick, 2008 ▶); program(s) used to refine structure: *SHELXL97* (Sheldrick, 2008 ▶); molecular graphics: *DIAMOND* (Brandenburg & Putz, 2005 ▶); software used to prepare material for publication: *SHELXL97*.

## Supplementary Material

Crystal structure: contains datablock(s) I, global. DOI: 10.1107/S1600536812021873/kp2415sup1.cif

Structure factors: contains datablock(s) I. DOI: 10.1107/S1600536812021873/kp2415Isup2.hkl

Additional supplementary materials:  crystallographic information; 3D view; checkCIF report

## Figures and Tables

**Fig. 1.**
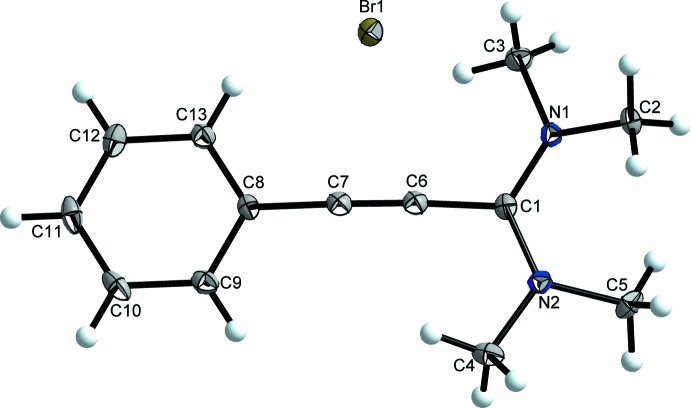
The crystal structure of N,N,N',N'- tetramethyl-3-phenyl-prop-2-yne-amidinium bromide with atom labels and 50% probability displacement ellipsoids.

## supplementary crystallographic information

### Comment

Acetylenic amidinium salts are characterised by a carbon-carbon triple bond, which is in conjugation with an amidinium function. They can be prepared by cleavage of alkyne orthoamides with triethylsilyltriflate or benzoyl chloride (Weingärtner *et al.*, 2011). Various alkyne orthoamides are transformed by elemental iodine or bromine to vinylogous guanidinium iodides or bromides (Kantlehner *et al.*, 2012). Phenyl substituted alkyne orthoamides like 3,3,3-tris(dimethylamino)-1-phenyl-prop-1-yne (Weingärtner *et al.*, 2011) behave differently, it reacts with bromine to give the title compound. According to the structure analysis, the C–N bond lengths in the amidinium unit are 1.325 (2) and 1.330 (2) Å, indicating double bond character. The positive charge in the cation is distributed between both dimethylamino groups. The bonds between the N atoms and the terminal *C*-methyl groups, all have values close to a typical single bond (1.462 (2)–1.465 (2) Å). The triple bond between C6 and C7 measures 1.197 (2) Å, with nearly linear C7–C6–C1 and C6–C7–C8 angles (176.3 (1) and 178.4 (1)°). The bond lengths between C7 and C8 as well as C1 and C6 are 1.427 (2) and 1.430 (2) Å, respectively. Similar geometric parameters have been observed in the crystal structure analysis of *N*,*N*,*N*',*N*',*N*'',*N*'',*N*''',*N*'''-octamethyl(but-2-yne)bis(amidinium)bis(tetrafluoroborate) (Drandarov *et al.*, 2012). The planes built up from the amidinium unit (N1, C1, N2) and the phenyl ring (C9, C8, C13) are twisted to each other by up to 10.3 (1)° (Fig. 1). Finally, no interactions between the cations and the bromide ions have been observed.

### Experimental

To a solution of 3,3,3-tris(dimethylamino)-1-phenyl-prop-1-yne (7.0 g, 28.5 mmol) in pentane (50 mL) was added dropwise a solution of bromine (4.56 g, 28.5 mmol) in pentane (50 mL) at 273 K with stirring. After 2 h stirring at ambient temperature the pale-yellow precipitate was filtered off *in vacuo* and recrystallised from acetonitrile; yield: 6.4 g (56%), pale-yellow single crystals. ^1^H NMR (60 MHz, CDCl_3_/TMS): d = 3.42 (s, 12 H, NMe_2_), 7.20–7.80 (m, 5 H, Ph–H).

### Refinement

Hydrogen atoms bound to aromatic carbon atoms were placed in calculated positions with d(C—H) = 0.95 Å and were included in the refinement in the riding model approximation, with *U*(H) set to 1.2 *U*_eq_(C). The hydrogen atoms of the methyl group were allowed to rotate with a fixed angle around the C–N bond to best fit the experimental electron density, with *U*(H) set to 1.5 *U*_eq_(C) and d(C—H) = 0.98 Å.

### Crystal data

**Table:**

C_13_H_17_N_2_^+^·Br^−^	*F*(000) = 576
*M**_r_* = 281.19	*D*_x_ = 1.408 Mg m^−^^3^
Monoclinic, *P*2_1_/*c*	Melting point: 441 K
Hall symbol: -P 2ybc	Mo *K*α radiation, λ = 0.71073 Å
*a* = 13.1009 (8) Å	Cell parameters from 4075 reflections
*b* = 10.6538 (6) Å	θ = 2.5–30.6°
*c* = 9.6611 (6) Å	µ = 3.08 mm^−^^1^
β = 100.276 (3)°	*T* = 100 K
*V* = 1326.81 (14) Å^3^	Block, yellow
*Z* = 4	0.28 × 0.20 × 0.15 mm

### Data collection

**Table:**

Bruker Kappa APEXII DUO diffractometer	4075 independent reflections
Radiation source: sealed tube	3466 reflections with *I* > 2σ(*I*)
Graphite monochromator	*R*_int_ = 0.032
φ scans, and ω scans	θ_max_ = 30.6°, θ_min_ = 2.5°
Absorption correction: multi-scan (Blessing, 1995)	*h* = −18→18
*T*_min_ = 0.483, *T*_max_ = 0.630	*k* = −15→15
27687 measured reflections	*l* = −13→13

### Refinement

**Table:**

Refinement on *F*^2^	Primary atom site location: structure-invariant direct methods
Least-squares matrix: full	Secondary atom site location: difference Fourier map
*R*[*F*^2^ > 2σ(*F*^2^)] = 0.021	Hydrogen site location: difference Fourier map
*wR*(*F*^2^) = 0.052	H-atom parameters constrained
*S* = 1.07	*w* = 1/[σ^2^(*F*_o_^2^) + (0.024*P*)^2^ + 0.4023*P*] where *P* = (*F*_o_^2^ + 2*F*_c_^2^)/3
4075 reflections	(Δ/σ)_max_ < 0.001
149 parameters	Δρ_max_ = 0.38 e Å^−^^3^
0 restraints	Δρ_min_ = −0.43 e Å^−^^3^

### Special details

**Table:**

Geometry. All e.s.d.'s (except the e.s.d. in the dihedral angle between two l.s. planes) are estimated using the full covariance matrix. The cell e.s.d.'s are taken into account individually in the estimation of e.s.d.'s in distances, angles and torsion angles; correlations between e.s.d.'s in cell parameters are only used when they are defined by crystal symmetry. An approximate (isotropic) treatment of cell e.s.d.'s is used for estimating e.s.d.'s involving l.s. planes.
Refinement. Refinement of *F*^2^ against ALL reflections. The weighted *R*-factor *wR* and goodness of fit *S* are based on *F*^2^, conventional *R*-factors *R* are based on *F*, with *F* set to zero for negative *F*^2^. The threshold expression of *F*^2^ > σ(*F*^2^) is used only for calculating *R*-factors(gt) *etc*. and is not relevant to the choice of reflections for refinement. *R*-factors based on *F*^2^ are statistically about twice as large as those based on *F*, and *R*- factors based on ALL data will be even larger.

### Fractional atomic coordinates and isotropic or equivalent isotropic displacement parameters (Å^2^)

**Table:**

	*x*	*y*	*z*	*U*_iso_*/*U*_eq_	
Br1	0.313905 (9)	0.523031 (12)	0.155779 (13)	0.01703 (4)	
C1	0.29358 (9)	0.46256 (11)	0.66879 (12)	0.0128 (2)	
N1	0.35589 (8)	0.56042 (10)	0.66379 (10)	0.01344 (19)	
N2	0.30756 (8)	0.37987 (10)	0.77285 (11)	0.0147 (2)	
C2	0.41450 (10)	0.62002 (12)	0.79005 (13)	0.0164 (2)	
H2A	0.3882	0.5904	0.8731	0.025*	
H2B	0.4065	0.7113	0.7823	0.025*	
H2C	0.4881	0.5982	0.7992	0.025*	
C3	0.35920 (11)	0.62655 (13)	0.53213 (13)	0.0205 (3)	
H3A	0.3140	0.5838	0.4546	0.031*	
H3B	0.4305	0.6271	0.5143	0.031*	
H3C	0.3353	0.7131	0.5392	0.031*	
C4	0.22431 (11)	0.29642 (12)	0.79900 (15)	0.0207 (3)	
H4A	0.1580	0.3255	0.7451	0.031*	
H4B	0.2210	0.2970	0.8995	0.031*	
H4C	0.2382	0.2109	0.7699	0.031*	
C5	0.40815 (10)	0.35338 (13)	0.86079 (14)	0.0203 (3)	
H5A	0.4635	0.3900	0.8175	0.031*	
H5B	0.4181	0.2624	0.8695	0.031*	
H5C	0.4103	0.3900	0.9543	0.031*	
C6	0.20698 (10)	0.44506 (11)	0.55744 (13)	0.0153 (2)	
C7	0.13169 (9)	0.42701 (11)	0.46956 (13)	0.0148 (2)	
C8	0.04099 (9)	0.40275 (11)	0.36723 (12)	0.0133 (2)	
C9	−0.02930 (10)	0.31159 (11)	0.39520 (14)	0.0170 (2)	
H9A	−0.0156	0.2651	0.4804	0.020*	
C10	−0.11893 (10)	0.28928 (12)	0.29838 (15)	0.0204 (3)	
H10A	−0.1669	0.2274	0.3171	0.024*	
C11	−0.13845 (10)	0.35719 (13)	0.17439 (14)	0.0203 (3)	
H11A	−0.2002	0.3420	0.1084	0.024*	
C12	−0.06867 (10)	0.44725 (13)	0.14572 (14)	0.0195 (3)	
H12A	−0.0828	0.4935	0.0603	0.023*	
C13	0.02160 (10)	0.46995 (12)	0.24136 (13)	0.0167 (2)	
H13A	0.0699	0.5308	0.2213	0.020*	

### Atomic displacement parameters (Å^2^)

**Table:**

	*U*^11^	*U*^22^	*U*^33^	*U*^12^	*U*^13^	*U*^23^
Br1	0.01516 (6)	0.01901 (6)	0.01757 (7)	0.00196 (5)	0.00471 (4)	−0.00012 (5)
C1	0.0109 (5)	0.0140 (5)	0.0139 (5)	0.0021 (4)	0.0035 (4)	−0.0027 (4)
N1	0.0132 (5)	0.0152 (4)	0.0115 (4)	−0.0012 (4)	0.0012 (4)	−0.0005 (4)
N2	0.0138 (5)	0.0147 (5)	0.0158 (5)	0.0012 (4)	0.0034 (4)	0.0016 (4)
C2	0.0149 (6)	0.0184 (6)	0.0150 (6)	−0.0022 (4)	0.0001 (4)	−0.0030 (4)
C3	0.0242 (7)	0.0219 (6)	0.0151 (6)	−0.0052 (5)	0.0028 (5)	0.0034 (5)
C4	0.0224 (7)	0.0158 (6)	0.0258 (7)	−0.0025 (5)	0.0091 (5)	0.0024 (5)
C5	0.0184 (6)	0.0224 (6)	0.0194 (6)	0.0077 (5)	0.0010 (5)	0.0047 (5)
C6	0.0148 (6)	0.0131 (5)	0.0182 (6)	0.0011 (4)	0.0034 (4)	−0.0018 (4)
C7	0.0140 (5)	0.0130 (5)	0.0179 (6)	0.0008 (4)	0.0045 (4)	−0.0020 (4)
C8	0.0105 (5)	0.0125 (5)	0.0167 (6)	0.0008 (4)	0.0023 (4)	−0.0039 (4)
C9	0.0162 (6)	0.0141 (5)	0.0216 (6)	−0.0001 (4)	0.0056 (5)	−0.0007 (4)
C10	0.0131 (6)	0.0171 (6)	0.0323 (7)	−0.0037 (5)	0.0076 (5)	−0.0081 (5)
C11	0.0105 (6)	0.0252 (6)	0.0246 (6)	0.0019 (5)	0.0010 (5)	−0.0124 (5)
C12	0.0171 (6)	0.0254 (6)	0.0153 (6)	0.0034 (5)	0.0009 (5)	−0.0022 (5)
C13	0.0140 (6)	0.0169 (6)	0.0191 (6)	−0.0015 (4)	0.0032 (5)	−0.0005 (5)

### Geometric parameters (Å, º)

**Table:**

C1—N2	1.3246 (15)	C5—H5A	0.9800
C1—N1	1.3304 (16)	C5—H5B	0.9800
C1—C6	1.4296 (17)	C5—H5C	0.9800
N1—C3	1.4615 (15)	C6—C7	1.1966 (17)
N1—C2	1.4651 (15)	C7—C8	1.4273 (17)
N2—C5	1.4625 (16)	C8—C13	1.3949 (17)
N2—C4	1.4636 (16)	C8—C9	1.3974 (17)
C2—H2A	0.9800	C9—C10	1.3851 (18)
C2—H2B	0.9800	C9—H9A	0.9500
C2—H2C	0.9800	C10—C11	1.384 (2)
C3—H3A	0.9800	C10—H10A	0.9500
C3—H3B	0.9800	C11—C12	1.3870 (19)
C3—H3C	0.9800	C11—H11A	0.9500
C4—H4A	0.9800	C12—C13	1.3860 (18)
C4—H4B	0.9800	C12—H12A	0.9500
C4—H4C	0.9800	C13—H13A	0.9500
			
N2—C1—N1	123.10 (11)	N2—C5—H5A	109.5
N2—C1—C6	117.91 (11)	N2—C5—H5B	109.5
N1—C1—C6	118.99 (11)	H5A—C5—H5B	109.5
C1—N1—C3	121.53 (10)	N2—C5—H5C	109.5
C1—N1—C2	122.91 (10)	H5A—C5—H5C	109.5
C3—N1—C2	115.04 (10)	H5B—C5—H5C	109.5
C1—N2—C5	123.89 (11)	C7—C6—C1	176.30 (13)
C1—N2—C4	121.91 (11)	C6—C7—C8	178.36 (13)
C5—N2—C4	113.86 (10)	C13—C8—C9	120.10 (11)
N1—C2—H2A	109.5	C13—C8—C7	120.69 (11)
N1—C2—H2B	109.5	C9—C8—C7	119.20 (11)
H2A—C2—H2B	109.5	C10—C9—C8	119.81 (12)
N1—C2—H2C	109.5	C10—C9—H9A	120.1
H2A—C2—H2C	109.5	C8—C9—H9A	120.1
H2B—C2—H2C	109.5	C11—C10—C9	119.90 (12)
N1—C3—H3A	109.5	C11—C10—H10A	120.0
N1—C3—H3B	109.5	C9—C10—H10A	120.0
H3A—C3—H3B	109.5	C10—C11—C12	120.53 (12)
N1—C3—H3C	109.5	C10—C11—H11A	119.7
H3A—C3—H3C	109.5	C12—C11—H11A	119.7
H3B—C3—H3C	109.5	C13—C12—C11	120.13 (12)
N2—C4—H4A	109.5	C13—C12—H12A	119.9
N2—C4—H4B	109.5	C11—C12—H12A	119.9
H4A—C4—H4B	109.5	C12—C13—C8	119.51 (11)
N2—C4—H4C	109.5	C12—C13—H13A	120.2
H4A—C4—H4C	109.5	C8—C13—H13A	120.2
H4B—C4—H4C	109.5		
			
N2—C1—N1—C3	160.73 (11)	C13—C8—C9—C10	−0.83 (18)
C6—C1—N1—C3	−19.49 (17)	C7—C8—C9—C10	178.38 (11)
N2—C1—N1—C2	−27.94 (18)	C8—C9—C10—C11	0.07 (18)
C6—C1—N1—C2	151.84 (11)	C9—C10—C11—C12	0.32 (19)
N1—C1—N2—C5	−25.57 (18)	C10—C11—C12—C13	0.06 (19)
C6—C1—N2—C5	154.65 (11)	C11—C12—C13—C8	−0.82 (19)
N1—C1—N2—C4	161.57 (12)	C9—C8—C13—C12	1.20 (18)
C6—C1—N2—C4	−18.20 (17)	C7—C8—C13—C12	−178.00 (11)
